# Telehealth and in-person HIV care during the COVID-19 pandemic at a large academic medical center in North Carolina

**DOI:** 10.1371/journal.pone.0320911

**Published:** 2025-06-04

**Authors:** Valerie Yelverton, Jan Ostermann, Michael E. Yarrington, Andrew K Weinhold, Nabil Natafgi, Bankole Olatosi, Sharon Weissman, Nathan M Thielman

**Affiliations:** 1 Department of Medicine, Division of Infectious Diseases, Duke University School of Medicine, Durham, North Carolina, United States of America; 2 Department of Health Services Policy and Management, University of South Carolina, Arnold School of Public Health, Columbia, South Carolina, United States of America; 3 South Carolina SmartState Center for Healthcare Quality (CHQ), University of South Carolina, Arnold School of Public Health, Columbia, South Carolina, United States of America; 4 Duke Global Health Institute, Duke University, Durham, North Carolina, United States of America; 5 Center for Health Policy and Inequalities Research, Duke University, Durham, North Carolina, United States of America; 6 Department of Internal Medicine, University of South Carolina, School of Medicine, Columbia, South Carolina, United States of America; 7 Prisma Health, Columbia, South Carolina, United States of America; Neurocrine Biosciences Inc, UNITED STATES OF AMERICA

## Abstract

**Background:**

To maintain HIV care during the COVID-19 pandemic, many HIV clinics across the United States adopted telehealth. However, not everyone participated in telehealth equally. This study assessed the use and disparities in telehealth and in-person HIV care at a large academic medical center in North Carolina (NC) relative to the COVID-19 pandemic.

**Methods:**

Data from the Duke University Infectious Disease clinic in NC were extracted from electronic health records (EHR), aggregated across persons with HIV (PWH) by calendar month, visit type (in-person vs. telehealth HIV care), and by key sociodemographic and clinical characteristics. Variation in HIV care over time was analyzed graphically by age, sex, race and ethnicity, county of residence, and viral load (VL) history.

**Results:**

EHR data from 2,623 PWH receiving care between January 2019 and March 2023 were included. Telehealth use sharply increased in the first months of the pandemic and decreased thereafter. Telehealth use was higher among non-Hispanic Whites compared to People of Color. Most PWH (93%) had a first post-onset-of-the-pandemic (*pop*) HIV care visit on March 16, 2020 and thereafter. The proportion of telehealth first *pop* visits peaked in April 2020 with 88% telehealth visits.

**Conclusions:**

Telehealth bridged the initial COVID-19 pandemic phase with drastically reduced in-person visit availability, yet it was not equally utilized across race and ethnicity groups. To guide the optimal integration of telehealth in HIV care and promote equitable care in the future, HIV care outcomes need to be closely monitored, and strategies designed to promote access for Communities of Color are needed.

## Introduction

During the COVID-19 pandemic, people with HIV (PWH) faced high levels of HIV care interruptions [1,2], with detrimental effects on HIV-related mortality [3]. In parallel with public health measures to limit the spread of COVID-19, telehealth was rapidly and widely implemented during the pandemic to mitigate HIV care interruptions, and offered by nearly all Ryan White-funded HIV care providers across the United States (US) [4]. Telehealth utilization increased sharply at the beginning of the pandemic and accounted for up to 90% of HIV care visits within the first half of 2021 [4–9]; most visits reverted back to in-person care shortly thereafter [10,11].

Telehealth use among subgroups of PWH varied by age, gender, race and ethnicity, injection drug use history, poverty, residence, housing instability, and clinical characteristics [6–8,11,12]. Research findings on HIV care visit adherence [5,10] and retention in care [4] remain conflicting. For example, Dawson et al. found that 38% of Ryan White-funded HIV care providers reported no changes in retention in care, 25% reported a slight improvement, and 28% reported a slight decline in retention in care [4]. Considering HIV-related laboratory assessment rates and outcomes, the evidence is similarly diverging [7–9,13]. While Spinelli et al. found significantly higher odds for PWH to have unsuppressed viral load outcomes after social distancing policies were enacted and increasing telehealth use was observed [8], other studies found no significant difference in viral suppression rates in their clinics associated with telehealth use [7,9]. Acknowledging the socio-demographic differences in the served client populations included in the above-mentioned studies, more evidence is needed to understand telehealth-associated changes in HIV care outcomes.

This study aims to provide insights into the utilization of telehealth and in-person HIV care at a large academic medical center in North Carolina (NC). Specifically, we examined variation in HIV care utilization and the use of telehealth HIV care over time and by race, ethnicity, age, sex, county of residence, and viral load (VL) history. We also analyzed variation in rates of first post-onset-of-the-pandemic (*pop*) HIV care visits (henceforth: first *pop* visits) by race, ethnicity, age, sex, county of residence, and visit type.

## Methods

### Design and ethics statement

A retrospective observational study design was used to characterize telehealth use among PWH. The study protocol was determined to be exempt from review by the Institutional Review Boards (IRBs) of the Duke University Health System (Pro00112920) and the University of South Carolina (Health Sciences South Carolina IRB; Pro00128577). The IRBs waived the requirement for informed consent.

### Setting and data

The study used electronic health records (EHR) data from the Duke University Infectious Disease (ID) Clinic in Durham, NC. NC ranked among the top ten states with the highest rates of new HIV diagnosis in 2021 [14]. The Duke ID Clinic is among the largest HIV care providers in NC and serves a heterogeneous patient population. Study data comprise all HIV care visits provided to adult PWH at the Duke ID Clinic between January 2019 to March 2023. The eligibility criteria for study inclusion are listed in the S1 File. EHR data from the Duke ID Clinic contained 18,154 visits provided to 2,623 PWH between January 2019 and March 2023 (averaging 336 visits per month).

### Data extraction

EHR data were extracted in June 2023 using Epic’s SlicerDicer tool (Epic, Verona, Wisconsin), a built-in reporting tool within Epic EHR management software. SlicerDicer can be used to generate data reports for predefined patient populations and measures (e.g., patients or visits). It allows for the custom filtering, or “slicing”, of EHR data for large clinic populations using sociodemographic and clinical categories. Visit-level data were aggregated across patients by calendar month and by key sociodemographic and clinical characteristics. For some variables, data aggregation required manualized steps, for example, some subcategories of covariates were generated by subtraction (see S1 File for details). Authors MY and AW had access to information that could identify individual participants during data collection. The authors documented the data in an aggregated manner that did not allow the identification of individual participants after the data retrieval.

### Variables of interest

The key outcome variable of interest, visit type, was defined as a binary variable describing either (1) an in-person visit or (2) a telehealth visit (including video and telephone visits). Covariates were defined as follows:

a. Time: calendar month of visit
b. Current age of PWH: 18–49 years old (henceforth: younger PWH) vs. 50 years or older (henceforth: older PWH) at the time of data extraction
c. County of residence: PWH who were living in the same county as the clinic location vs. PWH who were living in another county at the time of data extraction
d. Race and ethnicity: non-Hispanic White (henceforth: White) vs. non-Hispanic Black (henceforth: Black) vs. other PWH, including those whose race and ethnicity were unspecified (henceforth: other race and ethnicity)
e. Legal sex (as designated on governmentally issued identification documents): female vs. male vs. other
f. VL history in 2019: no VL test recorded in the EHR (henceforth: no VL test), all VL test results <200 copies/milliliter (c/ml; henceforth: fully suppressed, as defined by the Centers of Disease Control and Prevention [15]), vs. at least one VL test result ≥200 c/ml (henceforth: not fully suppressed)
g. VL history in 2022: no VL test, fully suppressed, vs. not fully suppressed (as defined above in VL history in 2019)

### Data analysis

Data were analyzed graphically to explore changes in the distribution of HIV care use, telehealth use, and first *pop* visits over time. The number of PWH seen per month through at least one in-person and/or telehealth visit were used to describe changes in visit rates and visit type over the course of the COVID-19 pandemic.

Monthly visit rates (technically describing rates of HIV care engagement across PWH) were calculated as the proportion of PWH with at least one in-person and/or telehealth visit that month out of the total number of PWH cared for at the Duke ID Clinic (N = 2,623). Monthly visit rates were calculated for all PWH (total monthly visit rates) and by covariates. Monthly rates of telehealth use were calculated as the proportion of PWH with at least one telehealth visit out of the total number of PWH with a visit that month.

First *pop* visits describe the first individual HIV care visit per person following the onset of the COVID-19 pandemic, including people new to the clinic and established patients. Monthly first *pop* visit rates (describing the rate at which PWH engaged in HIV care after the onset of the COVID-19 pandemic) were calculated as the proportion of PWH who had their first *pop* HIV care visit during each month on March 16, 2020 and thereafter out of the total number of PWH cared for at the Duke ID Clinic (N = 2,623). March 16, 2020 was selected because of institutional policy changes in response to the pandemic on this day. Low first *pop* visit rates are indicative of gaps in care after the onset of the pandemic. The proportion of PWH with telehealth first *pop* visits was calculated relative to the total number of first *pop* visits each month. The cumulative proportion of PWH with first *pop* visits regardless of the visit type (telehealth or in-person) was calculated as the sum of proportions across the present and previous months.

Disparities related to race and ethnicity, age, legal sex, county of residence, and VL history were defined as differences in visit, telehealth, or first *pop* visit rates per calendar month. Rates were calculated and visualized in Microsoft Excel (Microsoft Corporation, Redmond, WA).

## Results

### Sample characteristics

Table 1 summarizes sociodemographic and clinical characteristics of PWH cared for by the Duke ID Clinic during the study period. Most patients were aged 50 years or older (57%), non-Hispanic Black (58%), male (74%), and living in a county different from the ID clinic location (65%). The majority of patients had fully suppressed VL test results in 2019 (60%) and 2022 (62%). More than 9 in 10 PWH had a first *pop* HIV care visit (N = 2,425; 92% of the total patient population) between March 16, 2020 and March 31, 2023.

**Table 1:** Characteristics of people with HIV (PWH).

	Total PWH receiving care (January 1, 2019–March 31, 2023)	PWH with a first *pop* visit (March 16, 2020–March 31, 2023)
	N = 2,623	N = 2,425 (92.45% of total PWH)
**Current age**		
18-49 years old	1,123 (42.81%)	1,039 (42.85%)
50 years and older	1,500 (57.19%)	1,386 (57.15%)
**Race and ethnicity** ^a^		
Non-Hispanic White	728 (27.75%)	668 (27.55%)
Non-Hispanic Black	1,529 (58.29%)	1,427 (58.85%)
Other or unspecified	366 (13.95%)	357 (14.72%)
**Legal Sex**		
Female	672 (25.62%)	629 (25.94%)
Male	1,948 (74.27%)	1,793 (73.94%)
Other^b^	3 (0.11%)	3 (0.12%)
**County of residence**		
Clinic county	919 (35.04%)	857 (35.34%)
Other county	1,704 (64.96%)	1,568 (64.66%)
Viral load history in 2019^c^		
No viral load test	728 (27.66%)	
All < 200 c/ml	1,566 (59.50%)	Not assessed
At least one ≥ 200 c/ml	338 (12.84%)	
Viral load history in 2022^c^		
No viral load test	680 (26.17%)	
All < 200 c/ml	1,622 (62.43%)	Not assessed
At least one ≥ 200 c/ml	296 (11.39%)	

Percentages may not add up to 100 due to rounding. Abbreviation: PWH People with HIV; *pop* post-onset-of-the-pandemic; c/ml copies/milliliter.

^a^Frequencies reported in the ‘PWH with a first *pop* visit’ column show small discrepancies from the total number of patients and percentages do not add up to 100 due to selecting multiple categories and/or manual data aggregation.

^b^Due to the low number of PWH of other legal sex, their data are omitted in further analysis.

^c^N varies due to changes in total PWH served over the study period.

### HIV care services relative to the COVID-19 pandemic

**Fig 1:**
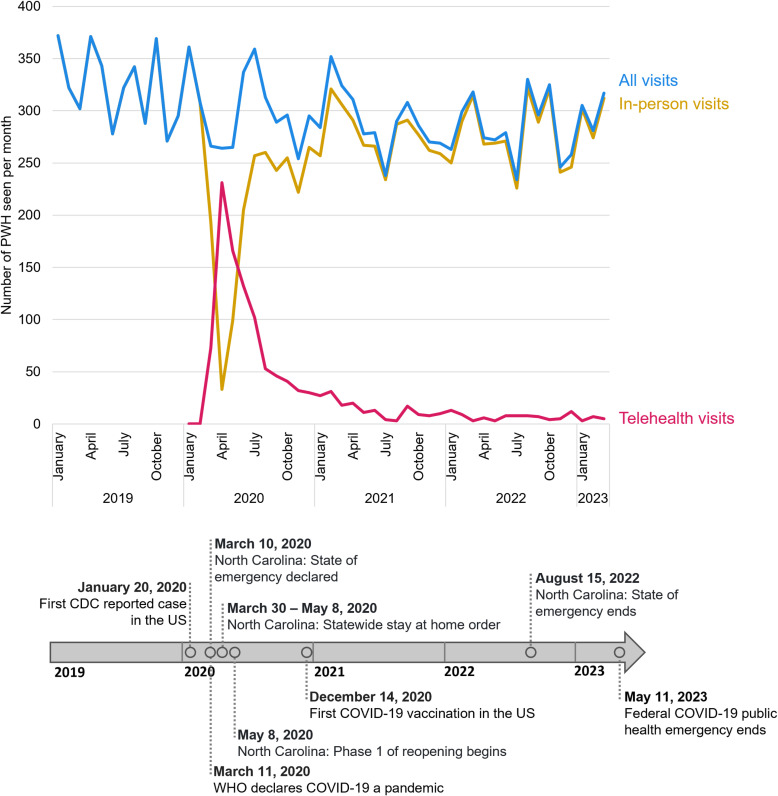
HIV care visit volume relative to the COVID-19 pandemic and pandemic-related events. Fig 1 details the number of PWH who received HIV care visits by visit type and calendar month and provides contextual information on COVID-19 pandemic-related events in the US and North Carolina during the study period. N = 2,623 PWH who received care at the Duke ID Clinic between January 2019 and March 2023. Pre-pandemic, all visits were provided in-person. COVID-19 pandemic-related events were retrieved from the CDC [16,17], the US Department of Health and Human Services [18], and the North Carolina General Assembly [19]. Abbreviations: CDC Centers for Disease Control and Prevention, US United States, PWH People with HIV.

In 2019, the number of patients seen at the Duke ID Clinic trended downwards (Fig 1). At the beginning of the COVID-19 pandemic (March to May 2020), the number of patients seen in-person each month dropped sharply and stabilized after 3 months at a lower level. Pre-pandemic, the Duke ID Clinic did not provide any telehealth HIV care visits. Telehealth was implemented in March 2020, in parallel with the COVID-19 pandemic and public health measures controlling the spread of COVID-19. Patients seen through telehealth surpassed the number of patients seen in-person in March, April, and May of 2020. After this phase, the number of patients seen through telehealth decreased and started leveling off in March 2021 at a rate of approximately nine patients receiving telehealth HIV care each month. The reduction in telehealth visits coincided with the end of the stay-at-home order and the beginning of the reopening phase in NC.

**Fig 2:**
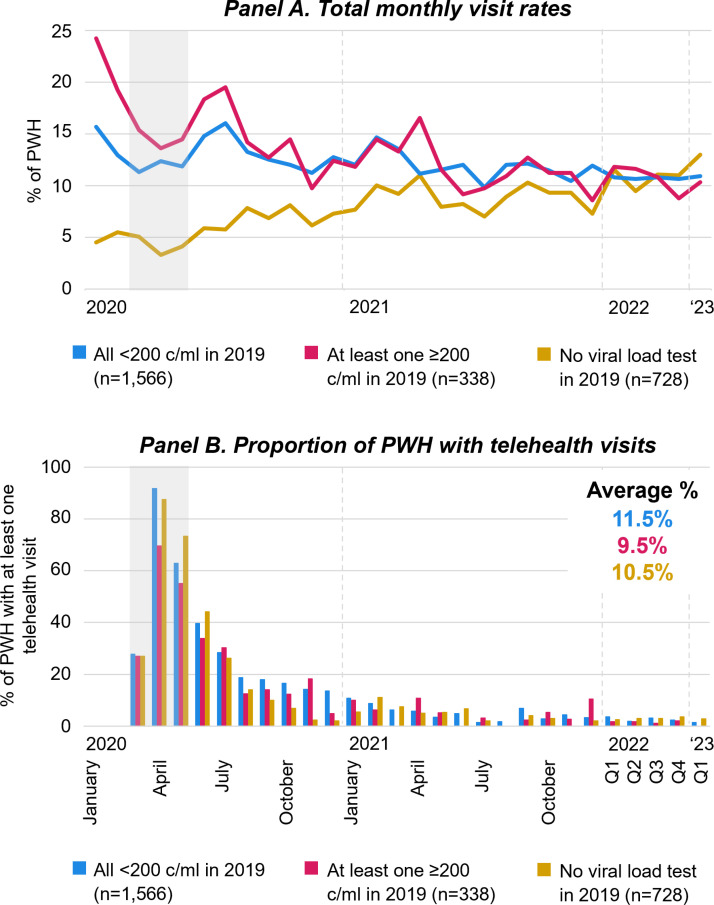
Engagement in HIV care in relation to the COVID-19 pandemic, by viral load history in 2019. Fig 2 displays monthly visits rates of PWH and the proportion of PWH who had at least one HIV care visit from January 2020 to March 2023 by VL history in 2019. Q1-4 refer to quarterly averages of the months within the quarter of the respective calendar year. Grey areas highlight March through May 2020 during which COVID-19 limitations on activities were most strict. Abbreviations: PWH People with HIV; c/ml copies per milliliter of blood.

**Fig 3:**
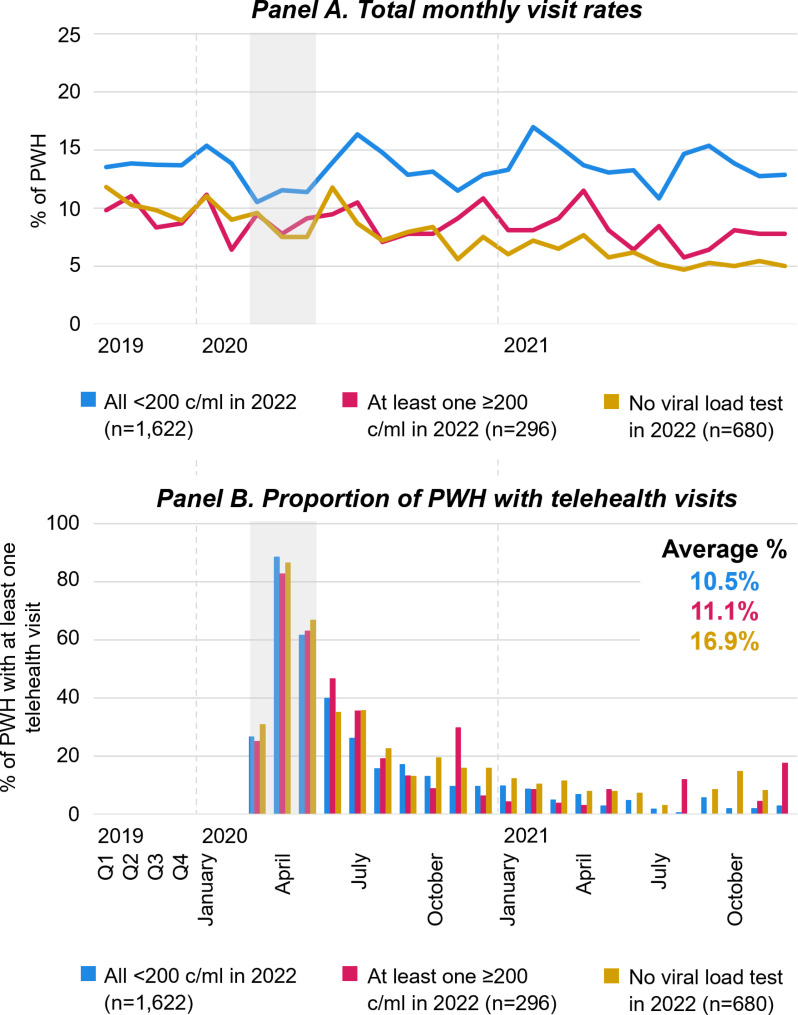
Engagement in HIV care in relation to the COVID-19 pandemic, by viral load history in 2022. Fig 3 shows monthly visits rates of PWH and the proportion of PWH who had at least one HIV care visit from January 2019 to December 2021 by VL history in 2022. Q1-4 refer to quarterly averages of the months within the quarter of the respective calendar year. Grey areas highlight March through May 2020 during which COVID-19 limitations on activities were most strict. Abbreviations: PWH People with HIV; c/ml copies per milliliter of blood.

Fig 2 and 3 describe variation in HIV care engagement and VL history relative to the COVID-19 pandemic. Pre-pandemic, the total monthly visit rate was highest among patients whose VL was not fully suppressed in 2019 (Fig 2, Panel A). Visit rate differences by 2019 VL test result decreased after the early pandemic in 2020. The proportion of patients who received at least one telehealth visit was highest among patients with fully suppressed viral load tests in 2019 and lowest among those whose VL was not fully suppressed (Fig 2, Panel B).

Comparing visit rates by VL history in 2022, total monthly HIV care visit rates were highest among patients whose VL was fully suppressed at all tests in 2022 (Fig 3, Panel A). Notably, for these patients visit rates rapidly recovered to pre-pandemic levels. Visit rates among patients whose VL was not fully suppressed in 2022 and those who had no VL test in 2022 show a decreasing trend throughout the study period. The proportion of patients with at least one telehealth visit was highest among those who had no VL test in 2022, followed by patients whose VL was not fully suppressed (Fig 3, Panel B).

**Fig 4:**
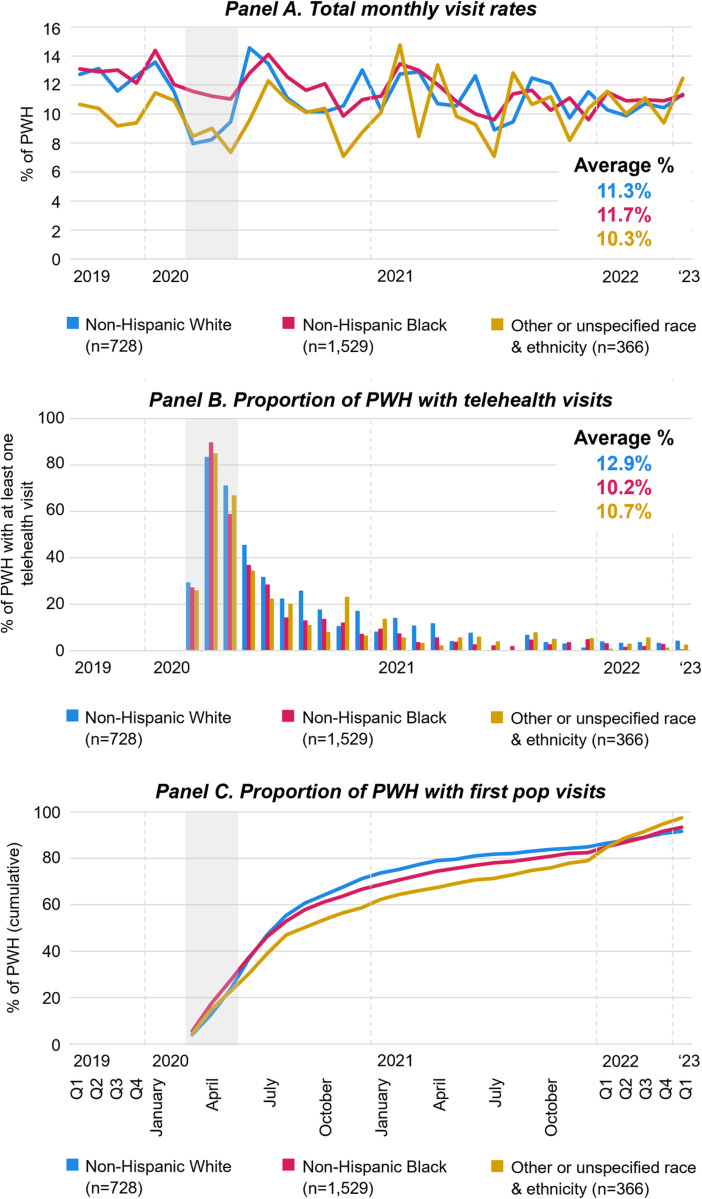
Engagement in HIV care relative to the COVID-19 pandemic by race and ethnicity. Fig 4 displays monthly visits rates of PWH (Panels A), the proportion of PWH who had at least one telehealth visit (Panels B), and the proportion of PWH with first *pop* HIV care visits (Panels C), by race and ethnicity from January 2019 to March 2023. For panels A and B, Q1-4 refer to quarterly averages of the months within the quarter of the respective calendar year; For panels C, Q1-4 refer to the end of the quarter cumulative proportion of people with HIV who had a first *pop* HIV care visit. Grey areas highlight March through May 2020 during which COVID-19 limitations on activities were most strict. Abbreviations: PWH People with HIV; *pop* post-onset-of-the-pandemic.

**Fig 5:**
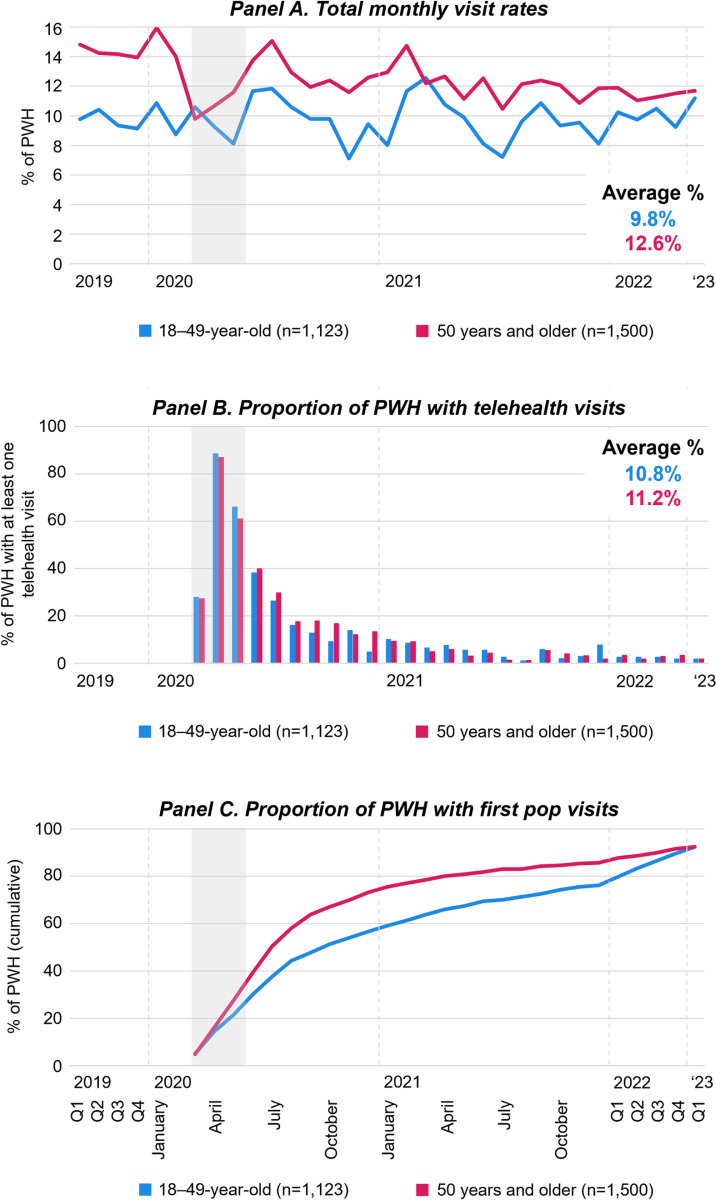
Engagement in HIV care relative to the COVID-19 pandemic by age. Fig 5 displays monthly visits rates of PWH (Panels A), the proportion of PWH who had at least one telehealth visit (Panels B), and the proportion of PWH with first *pop* HIV care visits (Panels C), by age from January 2019 to March 2023. For panels A and B, Q1-4 refer to quarterly averages of the months within the quarter of the respective calendar year; For panels C, Q1-4 refer to the end of the quarter cumulative proportion of people with HIV who had a first *pop* HIV care visit. Grey areas highlight March through May 2020 during which COVID-19 limitations on activities were most strict. Abbreviations: PWH People with HIV; *pop* post-onset-of-the-pandemic.

**Fig 6:**
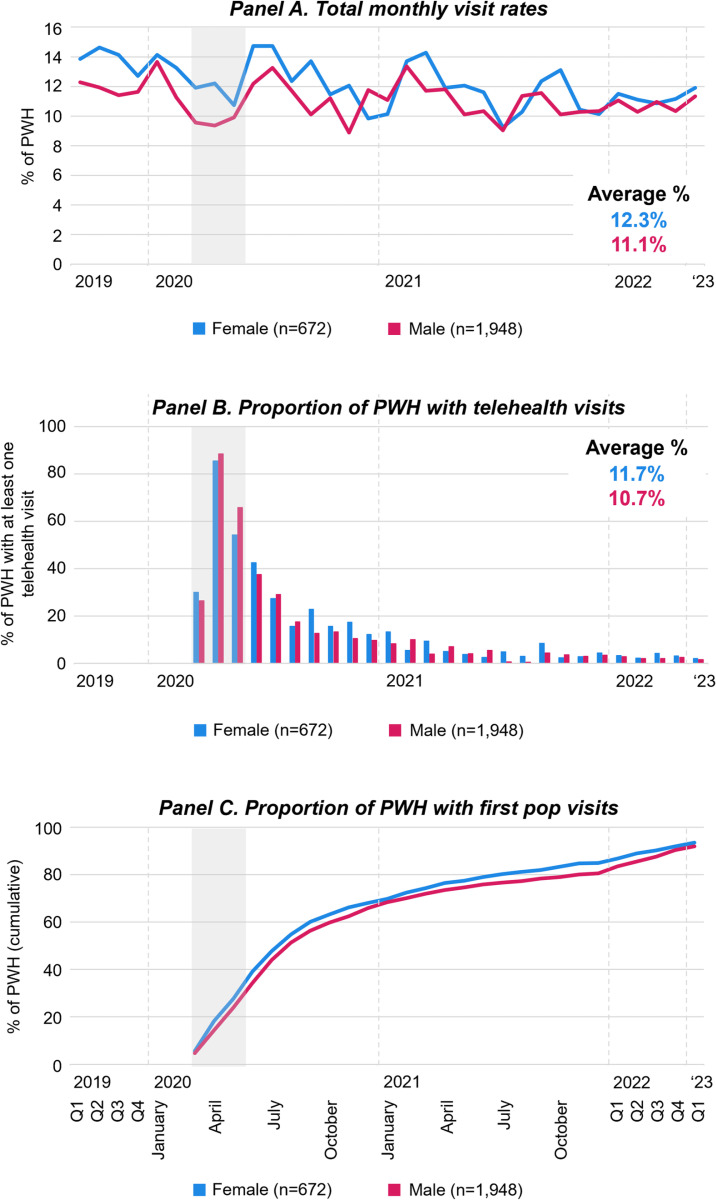
Engagement in HIV care relative to the COVID-19 pandemic by sex. Fig 6 displays monthly visits rates of PWH (Panels A), the proportion of PWH who had at least one telehealth visit (Panels B), and the proportion of PWH with first *pop* HIV care visits (Panels C), by sex from January 2019 to March 2023. For panels A and B, Q1-4 refer to quarterly averages of the months within the quarter of the respective calendar year; For panels C, Q1-4 refer to the end of the quarter cumulative proportion of people with HIV who had a first *pop* HIV care visit. Grey areas highlight March through May 2020 during which COVID-19 limitations on activities were most strict. Abbreviations: PWH People with HIV; *pop* post-onset-of-the-pandemic.

**Fig 7:**
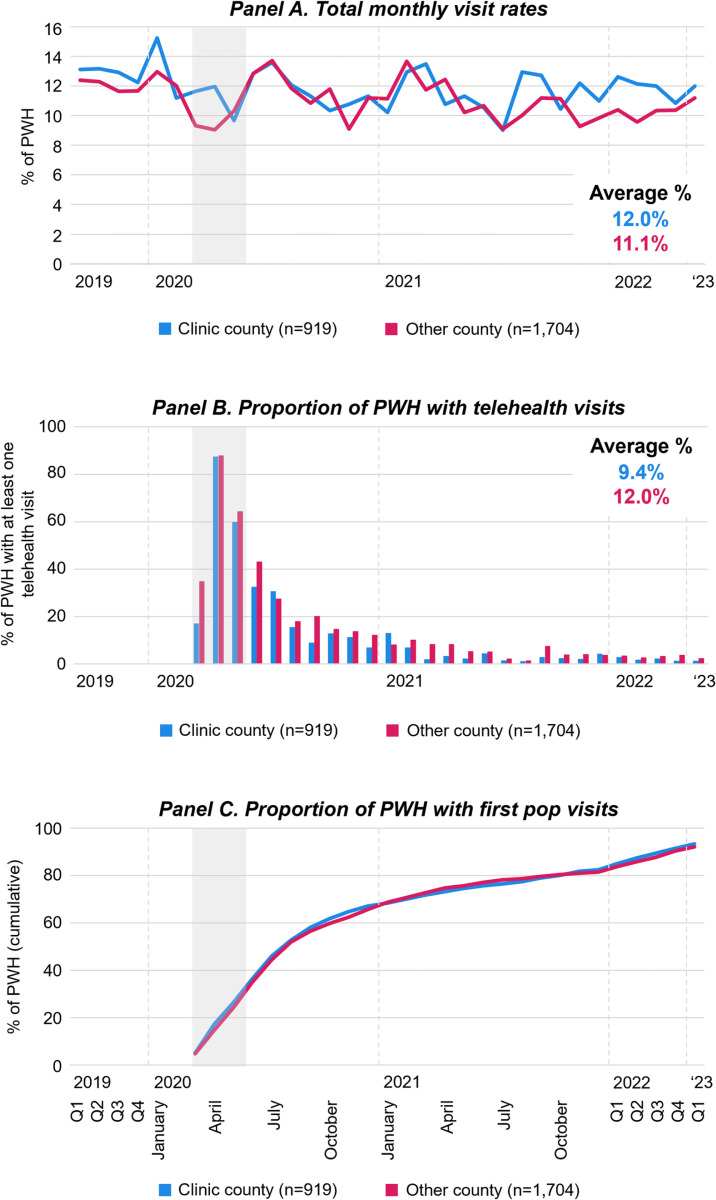
Engagement in HIV care relative to the COVID-19 pandemic by residence. Fig 7 displays monthly visits rates of PWH (Panels A), the proportion of PWH who had at least one telehealth visit (Panels B), and the proportion of PWH with first *pop* HIV care visits (Panels C), by residence from January 2019 to March 2023. For panels A and B, Q1-4 refer to quarterly averages of the months within the quarter of the respective calendar year; For panels C, Q1-4 refer to the end of the quarter cumulative proportion of people with HIV who had a first *pop* HIV care visit. Grey areas highlight March through May 2020 during which COVID-19 limitations on activities were most strict. Abbreviations: PWH People with HIV; *pop* post-onset-of-the-pandemic.

Figs 4–7 describe variation in HIV care engagement by sociodemographic characteristics. Fig 4 presents visit rates by race and ethnicity. The total HIV care visit rate trended downwards among White and Black patients; the pandemic-related decrease in visit rates was more pronounced for White patients (Panel A). The proportion of patients who received at least one telehealth visit was highest among White patients (Panel B). The cumulative first *pop* visit rate was lowest for White patients in the first months of the pandemic, but surpassed those of patients of other race and ethnicity in May 2020 and those of Black patients in July 2020 (Panel C).

Exploring age disparities in HIV care relative to the pandemic (Fig 5), showed that the HIV care visit rate among older patients is consistently higher that than the visit rates among younger patients (Panel A). The pandemic related decrease in the HIV care visit rate was more prominent in older patients reducing the distance between age-associated visit rates. The total visit rate among younger patients showed little change over time. Notably, for older PHW, visit rates in the first quarter of 2023 were substantially lower than those pre-pandemic. While younger PWH used telehealth at higher rates in the first three months of the pandemic, the proportion of patients who received telehealth visits was higher among older patients (Panel B). Older patients had higher first *pop* visit rates throughout the COVID-19 pandemic (Panel C).

In Fig 6, HIV care visit data is displayed by sex. The total HIV care visit rate pre-pandemic was slightly higher among females; yet, converged after the onset of the pandemic (Panel A). The proportion of patients who received at least one telehealth visit (Panel B) and cumulative first *pop* visit rates were slightly higher among female patients (Panel C).

Fig 7 presents HIV care visit data by residence. The total HIV care visit rates decreased at the beginning of the pandemic and stabilized at a slightly lower level at the end of 2020 regardless of residence (Panel A). The proportion of patients who received at least one telehealth visit was higher among PWH living in a county different from the HIV clinic (Panel B). Cumulative first *pop* visit rates were similar among patients regardless of residence (Panel C).

**Fig 8:**
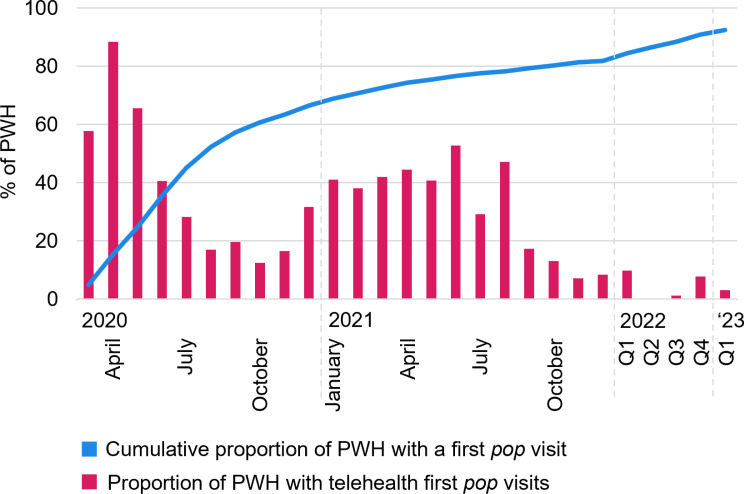
Engagement in first post-onset-of-the-pandemic (*pop*) HIV care visits and utilization of telehealth (N ** = 2,623).** Fig 8 shows the cumulative proportion of PWH who had a first *pop* visit (blue) and the proportion of PWH with telehealth first *pop* visits among monthly visit rates (red) after the onset of the COVID-19 pandemic in mid-March 2020. Q1-4 refer to the end-of-the-quarter cumulative proportion of PWH who had first *pop* visits or quarterly averages of telehealth rates among those PWH across months of the quarter of the respective calendar year. Abbreviations: PWH People with HIV; *pop* post-onset-of-the-pandemic.

Fig 8 displays engagement in first *pop* HIV care visits and utilization of telehealth. First *pop* visit rates increased most between March and August 2020; thereafter the cumulative trend flattened. The proportion of telehealth among first *pop* visits peaked at the beginning of the pandemic with 88% telehealth visits in April 2020. In parallel with the overall visit trends, telehealth use decreased thereafter; however, telehealth use for first *pop* visits showed a second peak in the first half of 2021.

## Discussion

This study is the first to assess in-person and telehealth HIV care utilization and disparities at a large academic medical center in NC relative to the COVID-19 pandemic. We identified variation in HIV care utilization over time by race and ethnicity, age, residence, and VL history. We further found differences in first *pop* visits by race and ethnicity, age, and visit type.

Our findings show that telehealth bridged HIV care interruptions at the beginning of the COVID-19 pandemic. While telehealth was utilized heavily initially, < 3% of the clinic's patients received telehealth in March 2021 and thereafter. This trajectory of telehealth use is reported in other literature [10,11,20], and coincides with COVID-19 related public health policies. By including pre-pandemic data we identified another noteworthy trend in HIV care utilization: the overall number of patients seen at this clinic declined after 2019. While telehealth compensated for the expected drop in in-person care visits at the beginning of the pandemic, it did not attenuate the overall downwards trend in HIV care utilization. The downwards trend stabilized in 2021. Potential factors attributing to this decreased monthly patient volume – though unlikely to fully explain it – may include increased utilization of multi-month prescriptions in response to COVID-19 [4] and potential changes in HIV care patterns. Future studies are needed to investigate the lower HIV care utilization.

Telehealth and in-person HIV care were not equally utilized relative to COVID-19. We found HIV care variation in age, race and ethnicity, and residence and provide new understanding to conflicting literature [6,7,11,12]. Regarding age, our analysis showed that older patients had higher overall HIV care engagement rates, higher telehealth utilization, and returned to HIV care earlier than their younger counterparts. These differences may stem from increased HIV and general medical care needs among older PWH who are at a higher risk of comorbidities [21]. Another factor that could contribute to the higher care engagement and earlier return to care among older PWH is work status. Older PWH may be retired and therefore more flexible in their schedules and time management [22]. Despite the relatively higher care utilization compared to younger patients, a post-pandemic decrease in overall visit rates was more prevalent among older patients as compared to their pre-pandemic HIV care visit patterns. Furthermore, telehealth did not compensate for the reduction in in-person care in the early pandemic. Higher risk for severe COVID-19 disease and outcomes [23], increased anxiety and other psychosocial challenges may have been more prevalent or severe among older PWH [24], and subsequently contributed to the accentuated changes in HIV care patterns pre- and post-pandemic.

The findings showed that White PWH used more telehealth than PWH of Color, mirroring other study findings [6,11]. However, notably, White patients returned to HIV care later than Patients of Color. First *pop* visit rates among White patients peaked in June of 2020, while rates among Black patients were highest in April of 2020. Delayed return to HIV care regardless of visit type after the onset of the pandemic as well as increased telehealth use among White PWH may be explained by sociodemographic differences as studies showed that gender, self-rated health status, and income were associated with delaying medical care during the COVID-19 pandemic [25,26]. In conjunction with data showing that People of Color and those with lower incomes participated in less work from home during the pandemic and thereafter [27], they may have experienced little change in their overall work and life pattern which is reflected in their return to care and in-person care use. Other mechanisms that may explain the delayed return to HIV care among White PWH include the end of the stay at home order in NC in May of 2020 [19] and a relative reduction in fear of COVID-19 and safety precaution among White people in parallel to rising knowledge of the drastic racial disparities of COVID-19 [28].

Patients living in a county different from the HIV clinic used telehealth at higher rates than patients who live in the same county as the clinic, which is in line with another study reporting higher odds for telehealth visit completion among people living farther from the HIV clinic [6]. As first *pop* visit rates were alike regardless of residence, telehealth appears to have substituted in-person visits suggesting that telehealth may help increase access to HIV care among PWH who live farther from the HIV clinic and may experience increased transportation and other access barriers to HIV care [29]. Future research is needed to optimize the allocation of telehealth to reach and meet the needs of those PWH who would benefit the most from telehealth and face higher access barriers to in-person HIV care.

Telehealth use also varied by prior viral load history. Telehealth was utilized more by patients whose VL was fully suppressed at all tests in 2019, which may be an indicator of treatment success and medication adherence. While the visit type decision process is not documented in EHR, it appears that telehealth was used predominantly for the care of established, clinically stable patients. These findings are supported by literature suggesting that clinical characteristics of PWH may have influenced HIV care visit type decisions during the COVID-19 pandemic and that telehealth is most suitable for established and clinically stable PWH [20,29].

Telehealth accounted for a particularly high proportion of first *pop* visits at the beginning of the pandemic and one year thereafter. While most studies focus on data from the earlier phase of the pandemic [5–8,10–13], this study incorporated important information on the use of telehealth as time progressed. The observed second peak in telehealth first *pop* visits suggests that telehealth continued to help linking PWH to care beyond the initial stages of the pandemic. Considering telehealth as a potential tool to mitigate HIV care access barriers, we hypothesize that this observed second peak in telehealth use for first *pop* visits may stem from PWH who did not return to HIV care earlier in the pandemic due to increased HIV care barriers. In future analyses, reasons and mechanisms affecting the timing and visit type choice of first *pop* visits are needed to evaluate this hypothesis.

Notably, overall monthly visit rates were highest and most stable throughout the pandemic among patients whose VL was fully suppressed at all tests in 2022. Lower overall visit rates among PWH whose VL was not fully suppressed in 2022 suggest that visit consistency and adherence may be more important for successful antiretroviral therapy than the visit type, and contribute new evidence to the conflicting findings on HIV care outcomes by visit type in the literature [7–9,13]. Patients who had no VL test in 2022 also showed lower and decreasing visit rates which may stem from PWH seeking care elsewhere. The relatively high proportion of telehealth visits among patients who had no VL test in 2022 may overlap with living further from the HIV clinic or the reported difficulty of integrating laboratory assessments within telehealth visits [20,30,31]. To overcome this disadvantage of telehealth, alternative strategies including the use of local laboratory facilities and home-based VL testing and monitoring [32] could be evaluated to guide the optimal integration of telehealth in HIV care and promote successful and equitable HIV care.

## Limitations

This study is subject to several limitations. First, EHR data used in this study came from one HIV care clinic in NC, which limits generalizability to PWH treated elsewhere. Second, we used aggregated data. Hence, patients with both in-person and telehealth visits within a calendar month (which anecdotally is relatively infrequent) would be counted double and people who have more frequent visits were counted in each calendar month they had a visit. People with multiple independent visits of the same visit type within one calendar month were counted once. To avoid counting people twice within multiple subcategories of time-varying covariates, we used age and county of residence at the time of data extraction rather than at the time of their visit. The number of people who had no VL test recorded in 2019 and 2022 are inflated by fluctuations in the patient panel cared for by the Duke ID Clinic. For example, people who have started care at the Duke ID Clinic in 2020 or thereafter are also recorded as people who had no VL test in 2019 even if they may have done testing at another health care system. In parallel, people who have transitioned out of care at the Duke ID Clinic before 2022 are recorded as not having any VL test in 2022 regardless of their potential care engagement elsewhere. Data aggregation related to first *pop* visits required multiple manualized steps increasing the data’s vulnerability to error. However, internal data review found low estimated error rates ranging from 2.0 to 2.7% which are not expected to change the findings of the study. Third, in-person and telehealth visit rate differences were assessed graphically rather than analytically. Findings from this study warrant further individual-level and multi-variable analyses to immerse deeper into telehealth disparities and explore causal relationships. Fourth, due to limited differentiation between types of telehealth in the EHR data, especially at the beginning of the pandemic, the visit type variable was defined as a binary variable combining video- and voice-only-based telehealth HIV care encounters.

## Conclusions

At a large academic center in NC, HIV care visit patterns changed relative to COVID-19. Although telehealth bridged the initial COVID-19 pandemic phase with drastically reduced in-person visit availability, it was not equally and consistently utilized throughout the COVID-19 pandemic. Telehealth use increased sharply in the early phase of the COVID-19 pandemic but reverted to predominantly in-person care one year later. PWH of Color utilized telehealth less than White patients indicating telehealth disparities in HIV care. To guide the optimal integration of telehealth in HIV care and promote equitable HIV care in the future, HIV care outcomes need to be closely monitored, and strategies designed to promote access for Communities of Color are needed.

## Supporting information

S1 File Additional methodological details. (DOCX)
