# Supplementary material for: Telehealth and in-person HIV care during the COVID-19 pandemic at a large academic medical center in North Carolina
Source: PLoS One. 2025 Jun 4;20(6):e0320911. doi: 10.1371/journal.pone.0320911 (PMC12136309; doi:10.1371/journal.pone.0320911)
Supplement: S1 File — (DOCX) [file pone.0320911.s001.docx]

**S1 File.** **Additional methodological details**

for manuscript “Telehealth and in-person HIV care during the COVID-19 pandemic at a large academic medical center in North Carolina.” by Yelverton, V et al.

**1. Eligibility criteria for HIV care visits**

The base patient study population was defined as

1) HIV-positive patients

2) seen by providers from any clinic within the Division of Infectious Diseases

3) at least once between January 2019 and March 2023

4) with an encounter type of Office Visit, Initial Consult, Telemedicine, or
Telemedicine-Phone.

**2. Detailed description of manualized data aggregation**

Six variables used in this analysis were manually aggregated due to functionality limitations of SlicerDicer or IRB restrictions. Under the exempt IRB protocol (Pro00112920), we were not allowed to export individual-level data and therefore manually documented data in Excel tables.

The subcategory ***all VL test results*** ***<200 copies/milliliter (c/m)*** of both covariates VL history in 2019 (subsection f. in the main manuscript section *Variables of interest)* and VL history in 2022 were (subsection g. in the main manuscript section *Variables of interest)* were calculated by subtraction. As there is no specific EHR field or category for VL test results below 200 viral copies per milliliter of blood, it was arrived at by subtraction of the other 2 categories from the total number of patients for that month by using the following formula:

$$Number of patients with all VL test results <200\frac{c}{ml} in a calendar month= Total number of patients seen in a calendar month- Number of patients with no viral load test in a calendar month-Number of patients with at least one VL test \geq200\frac{c}{ml}in a calendar month$$

The extraction of the data for the analysis related to **first *pop* visits** required manual identification and aggregation as our IRB approval prevented us from exporting individual-level data containing protected health information. As most patients had 1-5 visits in the study period after the beginning of the COVID-19 pandemic, we had to securely, manually review the data to exclude repeat visits by the same patient. Therefore, our data manager calculated overall reference numbers from SlicerDicer to use for confirmation. Our data manager further filtered the results and/or manually reviewed the data to tally the total number of in-person and telemedicine first *pop* visits and related patient characteristics. These steps were applied for the following variables: current age of PWH, county of residence, race and ethnicity, legal sex, VL history in 2019, and VL history in 2022.
